# Case report: Diagnostic challenges in an adolescent case of autistic catatonia

**DOI:** 10.3389/fpsyt.2024.1386949

**Published:** 2024-05-27

**Authors:** Nighat J. Nadeem, Abduallah Moawad, Sophie Howatson, Adeel Ahmed, Diana Cassell

**Affiliations:** General Adolescent Inpatient Unit, South West London and St George’s Mental Health National Health Service (NHS) Trust, London, United Kingdom

**Keywords:** autism spectrum disorder (ASD), catatonia, adolescents, CAMHS, neuropsychiatric, autistic catatonia

## Abstract

Catatonia is a complex neuropsychiatric syndrome involving a constellation of psychomotor disturbances including catalepsy, waxy flexibility, stupor, mutism, negativism, agitation, posturing, stereotypes, mannerisms, grimacing, echolalia, and echopraxia. Catatonia occurs in several conditions including psychotic, affective and neurodevelopmental disorders such as autism spectrum disorder (ASD). ASD is a neurodevelopmental disorder characterized by persistent deficits in communication, social interaction, restricted interests, repetitive behaviours and sensory sensitivities. Catatonia can occur in response to life stressors such as extreme fear or threat, interpersonal conflict, tragic events or following significant loss. Those with ASD may be particularly vulnerable to the negative impact of stressors and the link between catatonia and ASD is being increasingly recognized. The overlapping features of catatonia and ASD make it difficult to differentiate often resulting in delayed or missed diagnosis. Catatonia in ASD remains a significant clinical challenge; it is difficult to diagnose and can pose debilitating difficulties for those affected. Catatonia is a treatable condition and prompt recognition is vital in securing the best possible outcome. We report a complex and unique case of a 15-year-old boy who presented with severe cognitive and functional decline with a background history of significant bullying and deterioration in his mental state. This case posed a diagnostic conundrum leading to a diagnosis of underlying ASD, anxiety and trauma.

## Introduction

Catatonia was first defined by Karl Kahlbaum in 1874 and is a complex neurobiological disorder involving a wide spectrum of symptoms of motor, vocal and abnormal behaviours with impaired volition and vegetative function (1). DSM-5 defines catatonia by the presence of at least three of the following symptoms: catalepsy, waxy flexibility, stupor, mutism, negativism, agitation, posturing, stereotypes, mannerisms, grimacing, echolalia, and echopraxia (2). It has also been defined as a ‘marked decrease in reactivity to the environment’ (3) and in its most severe form, ‘malignant or lethal’ catatonia, it can cause serious complications including pneumonia, decubitus ulcers, thrombosis, malnutrition, dehydration, rhabdomyolysis and consistent mortality rates (4). Catatonia is reported to be the most severe psychiatric condition as it increases the risk of premature death (including suicide) by 60-fold (5). Catatonia can occur in several conditions including psychotic, affective and neurodevelopmental disorders as well as physical medical conditions (6). The link between autism spectrum disorder (ASD) and catatonia is being increasingly reported in the literature and this is reflected by a recent increase in publication trends on this topic (7). The term ‘autistic catatonia’ has been used and is defined as ‘freezing when carrying out actions, resistance to prompting, slow voluntary motor movements, and stopping in the course of movement’ (8).

ASD was first described by Eugen Bleuler in 1911 and is a neurodevelopmental disorder characterized by persistent deficits in communication, social interaction, restricted interests, repetitive behaviours and sensory sensitivities (2). The estimated prevalence of ASD is 1 in 54-59 children, and is 4.3 times more prevalent in boys than in girls (9, 10). Comorbidities are common in ASD and include emotional, behavioural, somatic disorders including epilepsy, gastro-intestinal disorders or sight/hearing impairments (11). Studies estimate the prevalence of at least one comorbid psychiatric disorder at 54.8% and up to 94%, with Attention Deficit Hyperactivity Disorder (ADHD), anxiety, depressive disorders and sleep disorders being the most frequent (12).

Research suggests that catatonia affects 0.6%-17% of child and adolescent psychiatric inpatients (13–15) and in ASD catatonia has a peak age of onset between 15-19 years and occurs more commonly in boys (70-100%) (16). Approximately 12-17% of people with ASD develop catatonia (17) and it is likely that the true prevalence of catatonia in autistic children is likely to be higher than reported given the varying presentation and low index of suspicion amongst clinicians (18). The aetiology of catatonia in ASD is not clearly understood with several hypothesis regarding the pathophysiology including neurotransmitter, genetic, metabolic abnormalities and psycho-sociological factors such as trauma and severe stress (18, 19). Some patients may be genetically predisposed to developing catatonia with genes on chromosomes 15 and 22 being linked to periodic forms of catatonia (8).

Environmental stressors are recognized as a risk factor in the development of catatonia, and autistic young people may be particularly vulnerable to the impact of these stressors. Adolescence is a transition period of ‘storm and stress’ (20) and is a time with significant psychological, physiological and social change. At times, it can be difficult to decipher which aspects of a presentation relate to the process of normal adolescent development. Autistic adolescents might have specific vulnerabilities which may make them prone to displaying a heightened negative response to adverse events as compared with neurotypical peers. Studies have suggested that people with ASD may be particularly vulnerable to intense fear and display an elevated cortisol in response to stress (21–23). Trauma is a known risk factor for the development of catatonia and literature suggests catatonia can occur in response to extreme fear, interpersonal conflict, a tragic event, abuse or following significant loss (18). One of the main daily stressors in young autistic people is bullying (24) and those with ASD may be predisposed to develop a heightened response to trauma given their particular sensitivities, need for routine and predictability. In such ways, disruption to routines, loss and stressors can cause significant distress. Therefore, in ASD catatonia may be an evolutionary response and coping strategy to extreme fear, and present as an expression of extreme anxiety (25).

Catatonia remains underrecognized and undertreated among young people (19). Although it is treatable when promptly identified (6), its heterogeneous presentation and overlapping features with other conditions poses a clinical challenge and can lead to missed diagnosis and missed treatment opportunities. We report a complex and unique case of a 15-year-old boy who presented with severe cognitive and functional decline with a background history of significant bullying and deterioration in his mental state. This case posed a diagnostic conundrum leading to a diagnosis of underlying ASD, anxiety and trauma. This case caused diagnostic uncertainty and raised pertinent clinical questions requiring significant MDT input to reach a consensus on the diagnosis.

## Case presentation

X is a 15-year-old boy originally of Pakistani origin who presented to mental health services following a period of progressive and significant deterioration in his mental state, decline in his cognitive abilities and overall functioning. He also presented with sexual disinhibition, odd behaviours and an inability to independently attend to his activities of daily living (ADL).

X was born in Italy three weeks prematurely via planned caesarean section as his mum had already had two previous caesarean sections. He developed breathing difficulties when he was three days old and required a two month admission to hospital. He achieved all his developmental milestones on time and there were no concerns regarding his development. X spent his early years in Italy and went to nursery there. It was noted that he did not interact much with his peers and it was felt that this was likely due to the language barrier. X moved to the UK in 2015 with his family when he was 7 years old. At this time, he was only able to communicate in Urdu and did not learn to speak Italian or English. Once at school in the UK, X rapidly developed good English language skills. His level of spelling and reading was noted to be above average and there were no concerns about his academic potential or learning ability.

X is the youngest of four children. He lives with his parents and grandparents, all of whom have physical health conditions and limited mobility. His mother had recent surgery for suspected bone cancer and his father has back problems and depression. Both have limited mobility and require the use of a walking stick. There is no known family history of ASD. His past medical history includes asthma and egg allergy.

X would spend most of his time at primary school with his older brother who is 2 years older than him. X was bullied in year 7 of secondary school and his mental health improved with school closures during the COVID-19 pandemic. On his return to school after the lockdowns in September 2021, X struggled to adjust and cope with the transition, and this is when his difficulties became apparent and noticeable.

In October 2021, X was the victim of severe and significant bullying (physical and sexual), and he was pushed against a metal object by a peer resulting in him banging his head. He required 15 stitches for the injury, but his GCS was 15/15 and CT head was normal. He did not require admission or follow up and this head injury did not cause any physical damage to his brain.

In March 2022, X appeared low in mood, withdrawn and was referred to his local CAMHS team. A month later, X began to display odd behaviors such as drinking lotions and collecting tissues in baskets. He was also seen to be typing messages in a stereotypic manner. X became increasingly anxious, socially withdrawn, had disturbed sleep, was less communicative and refused to leave home. He appeared low in mood, apathetic and seemed to have difficulty understanding and retaining information. From June-July 2023, X had multiple presentations to A&E for aggressive behaviour at home. Given the continued deterioration in his mental state, in July 2022 X had an MRI brain which showed no intracranial pathology other than non-specific white matter changes suggestive of periventricular leukomalacia (PVL), likely related to being born prematurely.

X remained under the care of the Adolescent Outreach Team from 10 July 2023 with home visits twice per week and daily phone calls to parents. X was started on Risperidone and titrated up to maximum 2mg once daily and Lorazepam 0.5 mg three times a day on 10.07.23. Despite this treatment and input, X showed little sign of improvement in his mental state or functioning. He spent most of his time in bed and was unable to manage his personal hygiene. His appetite reduced significantly, and he would only eat crisps. X was communicating significantly less than he usually would with his family and mostly responded by saying ‘I don’t know’. He became reluctant to allow his parents to assist him with his physical needs and would become physically aggressive towards them by hitting and kicking. X developed suicidal ideation and told his parents he did not want to live anymore. Given his mental state and level of functioning, it was felt that X’s needs could not be met in the community as he was requiring significant nursing care for his ADLS. This posed additional pressures on his parents given their own physical health needs.

In July 2023, X was admitted to a general adolescent inpatient ward where he presented as calm, minimally verbal responding to all questions with ‘I don’t know’ and had poor short term and long term memory. X was unable to follow visual aids and reminders as he would seem to forget what to do next. For example, he would go into the shower but would appear ‘frozen’ despite prompts being given by staff. As an inpatient, X was supported with psychology and occupational therapy, however he engaged very minimally with staff and peers on the ward, mostly responding with ‘I don’t know’. Differential diagnosis on admission included first episode psychosis, autoimmune pathology, infective/metabolic organic conditions, neurogenetic conditions, catatonia and ASD. X did not exhibit any signs of psychosis; therefore, Risperidone and Lorazepam were stopped. There was no deterioration in his mental state following the stopping of these medications.

In collaboration with the Paediatric Neurology team, he had extensive investigations including routine and specialist metabolic blood tests, EEG and MRI brain. Other than a mild anaemia, all these investigations were normal. Therefore, an underlying organic cause of X’s difficulties had been ruled out. X’s case was also discussed with the specialist CAMHS Learning Disabilities team, and it was concluded that X does not have a learning disability. However, it was noted that there were features in his medical notes that may be suggestive of ASD. Therefore, X was assessed for ASD and based on a detailed developmental history and observations of X on the ward, it was felt that X met the threshold for a diagnosis of ASD. The consensus from all professional teams involved was that X’s difficulties were likely due to autistic catatonia, and so X was started on Lorazepam and titrated up to 1mg four times a day. Soon after, there was a noticeable and significant change in his mental state as he became more communicative and outspoken. He began to speak about feeling anxious, unsafe in the ward environment and recalling memories of being bullied. Initially it seemed that he was showing a good response to Lorazepam as he was less catatonic in presentation. It was felt that X was suffering from anxiety, so he was started on Sertraline and titrated up to 150mg once daily. After this initial good response to Lorazepam, X became verbally and physically aggressive as well as sexually disinhibited. He required frequent seclusion on the ward as he was displaying inappropriate behaviours such as touching himself inappropriately in communal areas and attempting to touch staff/peers inappropriately.

**Figure 1:**
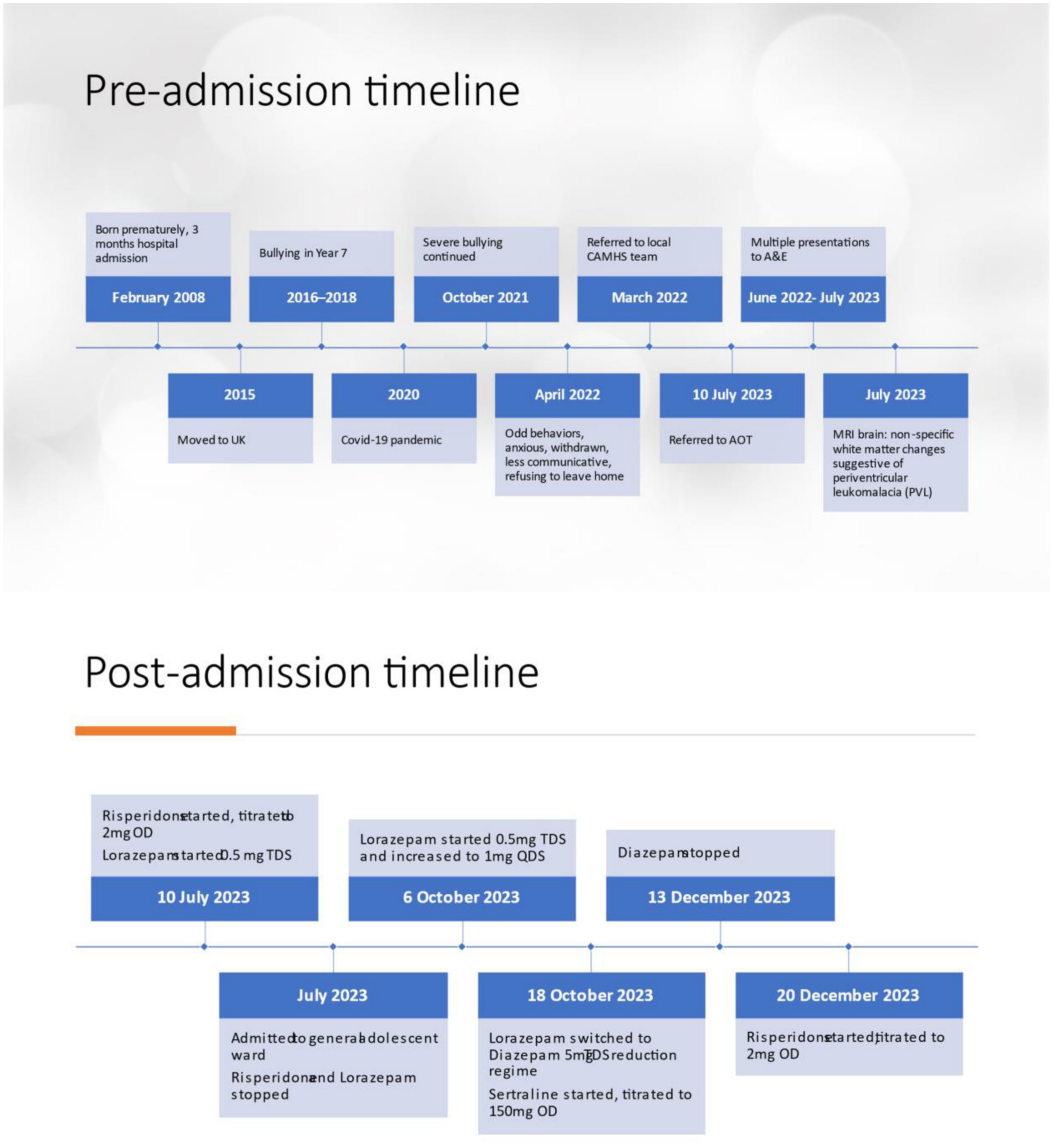
Timeline.

Given the coincidental timing of X starting Lorazepam and him displaying significant disinhibited behaviours, it was decided that X may have been having a paradoxical reaction to Lorazepam which was then switched to Diazepam, weaned down and stopped on 13.12.23. The disinhibited behaviours reduced, however he continued to display some aggressive and disinhibited behaviours to a lesser extent. X’s parents reported that they were struggling to manage his behaviours at home when he would be sexually inappropriate towards his family members. Given X’s difficult behaviours, on 20.12.23 he was started on Risperidone and titrated up to 2mg which had a good effect. Towards the end of his inpatient admission, X was able to engage better with the ward team and benefit from structured activities. He was able to respond to staff’s prompts and use visual aids to independently do activities. Discharge was planned to include a package of care. His pre and post admission timeline of events are summarised in **Figure 1**.

This case is a good example of the significant clinical challenges posed by the entangled features of ASD, catatonia, anxiety and trauma. On discharge, X’s diagnosis was ASD, catatonia, anxiety and trauma. His medication regime on discharge was Sertraline 150mg once daily and Risperidone 2mg once daily.

## Discussion

This case describes catatonia in a 15-year-old boy who presented in a complex and unclear manner. The initial presenting clinical picture had features of several differential diagnoses which led to diagnostic uncertainty and raised pertinent clinical questions requiring significant MDT input to reach a consensus on the diagnosis. After extensive monitoring, assessment and investigation the consensus of all professional teams involved was a diagnosis of ASD, anxiety and trauma.

In X’s case, the possibility of ASD had not been raised as a concern throughout his primary school years. It is likely that as the youngest of four siblings, he had been well scaffolded and supported by his family and was able to manage the social demands placed on him during his younger childhood years. The transition from primary to secondary school is a landmark moment in a child’s life (26) and it is not unusual for children to begin to struggle once they move to secondary school. Autistic children are known to be particularly vulnerable during this important transition phase. Evidence suggests that autistic children have negative experiences and struggle to adjust to secondary school (27–29). There may be several reasons for this including adapting to a new physical environment, new daily timetable, new teachers, new peers and the increased social demands may exceed the child’s capacity to cope.

There is a global increase in ASD prevalence and there is substantial clinical burden associated with ASD (30). It is usually diagnosed in the pre-school years, however a diagnosis in later years is not uncommon as in X’s case. It is known that children who are diagnosed with ASD later often have high levels of comorbid mental health and social difficulties (31). In X’s case, the overlapping comorbid features made it difficult to differentiate and tease apart and it is well known that psychiatric and somatic comorbidities complicate the evaluation, management and prognosis of ASD (30). Comorbidities and the associated complexities can lead to misdiagnosis, missed diagnosis and delayed diagnosis contributing to inadequate management and potentially missed opportunity for appropriate management.

The pathogenesis of autistic catatonia remains elusive and there are a multitude of hypothesis including neurotransmitter, genetic, metabolic abnormalities and psycho-sociological factors such as trauma and severe stress (18, 32). Autoimmune activation has been associated with both ASD and catatonia (33, 34) and autoimmune encephalitis should be considered in any patients with neurodevelopmental disorders presenting with catatonia (7). This was thoroughly investigated and excluded in X’s case and the aetiology remains unclear and likely multifactorial. It is reported that irrespective of the underlying cause of catatonia in ASD, eliminating the trigger and prompt medical management is essential to prevent complications (35).

One significant trigger in X’s case is the history of significant and severe bullying. Given the link of autistic catatonia with stress and trauma, post-traumatic stress disorder (PTSD) and trauma related psychological therapy was considered. The impact of traumatic events on any child’s mental health can present itself in a variety of ways. It is known that often in response to trauma, victims can display a freeze response which is likely to be a primitive evolutionary response to danger. Catatonia may be thought of as an extreme reaction to stress as exposure to life stressors including physical abuse have been linked to the emergence of catatonia in autistic people (18, 36). Although there are no clear guidelines on the evaluation of trauma in catatonia, it is worth considering screening patients with neurodevelopmental disorders presenting with catatonic symptoms for recent stressors or traumas to aid diagnosis and potentially guide treatment (7).

Given the overlap in symptomatology with ASD and catatonia, there appears to be common neurobiological mechanisms underlying the pathogenesis and improving our understanding of these would go some way to help make sense of such complex presentations where catatonic and ASD features are both present (14). A suggestion of one way to differentiate between ASD and catatonia is that symptoms in catatonia are typically new onset usually in late adolescence (37). This is similar to X’s case as he developed new onset of difficulties in his teenage years. There are 40 different symptoms of catatonia (38) and reports suggest that it is often underdiagnosed, and considered “hidden in plain sight” amongst other disorders (39). In X’s case mutism and being minimally verbal was a noticeable change, and a large study exploring the multiple ‘faces’ of catatonia in ASD suggest that 73% displayed mutism (36). There are also suggestions that mutism alone may predict impending catatonia in children with ASD (18), and is a key feature to look out for. With hindsight based on our experience of caring for X, we feel it is important to consider the possibility of catatonia in a child displaying a significant departure from their baseline in terms of speech and language as well as a regression of other previously acquired functioning skills.

X displayed increased disinhibited behaviours after starting benzodiazepines and the literature suggests that catatonic patients with ASD may be less responsive to benzodiazepines (16). Once these were stopped, X appeared to have benefited from atypical antipsychotic medication and it is known that low dose antipsychotics have weak GABA agonist activity and serotonin antagonism that could stimulate dopamine release in the pre-frontal cortex and alleviate catatonic symptoms (37). The antipsychotic medication may have helped to manage X’s difficult behaviours most likely related to ASD and have allowed him to be successfully discharged from the ward.

This case report adds to the existing evidence that the presence of an underlying undiagnosed ASD should be considered in any individual presenting with catatonia (14) and a decline in functioning and deterioration in previously acquired skills in young people should prompt consideration of the possibility of catatonia. Catatonia in ASD is more common than previously recognised and proposals have been made to screen all patients with neurodevelopmental disorders with screening tools to diagnose and treat them appropriately (40). The Paediatric Catatonia Rating Scale (PCRS) is the most reliable scale to assess for catatonia in children and is validated for paediatric patients in the inpatient setting (41).

There remain significant gaps in awareness, knowledge and understanding about autistic catatonia. Further work is required to refine the wide cluster of clinical features and validate diagnostic criteria for autistic catatonia. We suggest building on recent advances in this area to further improve our understanding of the interlinked neuropathological processes in ASD and catatonia. This would help in understanding the role of environmental stressors, identify autistic young people who are more susceptible to developing catatonia as well as to identify prognostic indicators.

### Parents perspective

Parents describe X as being ‘intelligent and active’. He would do well in school and play sports such as table tennis and chess. He would also be socially active and engage in conversation with family and friends. X would easily become friendly when meeting new people. X’s parents feel that he was over-sensitive and he would get hurt. X would feel that other people are being unkind, teasing and ignoring him when they had not intended to do so. X was also very caring and would always be willing to help others. He would be happy to give his pocket money to beggars on the street. X’s father remembers that X would not like it if his father did not give money to beggars or put money in donation boxes. X would insist that his father donate money and would tell his dad that he is ‘not doing a good thing’ by not donating.

X’s parents describe him as being ‘loved more’ as he was born prematurely and was very unwell soon after birth. X’s parents feel that X is the ‘youngest child in the house and will always be the youngest child’. In this way, as a family they have supported X and not place demands on him. X would struggle to understand things and became more insistent that things were done his way and his opinions are correct.

Parents feel that throughout his early years, X was developing normally and not any different to their other children so they did not have any concerns. His school teachers also did not raise any concerns. Parents feel that X’s difficulties started when he was bullied at school and the trauma related to this is a significant factor in X’s difficulties and are hopeful that he will make a good recovery.

## Conclusion

This case report is a unique and rare presentation of autistic catatonia and adds to the existing literature about adolescent cases of autistic catatonia and contributes to raising awareness in the medical community about this important yet all too easy to miss diagnosis. Delayed diagnosis of autistic catatonia increases the likelihood of complications as well as posing significant morbidity for the patient and burden for their family and the healthcare system. Through this case report, we aim to raise awareness, suggest a need for screening as it is difficult to diagnose, and maintain a high index of suspicion to allow for timely detection. This is vital to ensure timely recognition and minimize barriers to these young people receiving optimum care. From our experience we advise maintaining a cautious and thoughtful approach to avoid falling into the trap of diagnostic overshadowing in such presentations which have an entangled and complex course.

## Data availability statement

The original contributions presented in the study are included in the article/supplementary material. Further inquiries can be directed to the corresponding author.

## Ethics statement

Written informed consent was obtained from the minor(s)’ legal guardian/next of kin for the publication of any potentially identifiable images or data included in this article.

## Author contributions

NN: Conceptualization, Data curation, Methodology, Project administration, Resources, Software, Supervision, Visualization, Writing – original draft, Writing – review & editing. AM: Writing – review & editing. SH: Writing – review & editing. AA: Writing – review & editing. DC: Conceptualization, Writing – review & editing, Supervision, Visualization.
